# Effect of framed mHealth messages on oral hygiene and quality of life among Sudanese refugees in Egypt: a randomized controlled trial

**DOI:** 10.1186/s12903-026-09252-z

**Published:** 2026-07-17

**Authors:** Dina Attia, Mona K. ElKashlan, Susan M. Saleh, May M. Adham

**Affiliations:** Department of Pediatric Dentistry and Dental Public Health, Faculty of Dentistry, Alexandria University, Alexandria, Egypt

**Keywords:** Message framing, Plaque index, Gingival index, OHRQoL, Oral hygiene, Refugees, Egypt

## Abstract

**Background:**

Sudanese refugees in Egypt face substantial barriers to oral healthcare access, resulting in high disease burden and poor oral health–related quality of life (OHRQoL). This study evaluated impact of gain- and loss-framed mobile text messages (mHealth) on oral health status, OHRQoL, and hygiene practices among this population.

**Methods:**

A randomized controlled parallel trial was conducted between November 2024 and December 2025 among Sudanese refugees in Alexandria, Egypt, using snowball sampling. Eligible participants (≥ 18 years) were randomly allocated (1:1:1) into gain-framed SMS (emphasizing benefits), loss-framed SMS (emphasizing consequences), or control groups. Intervention groups received 24 SMS messages over 12 months. Outcomes were assessed at baseline, 6 months, and 12 months using clinical examinations (Plaque Index and Gingival Index), OHIP-5 questionnaire for OHRQoL, and a self-reported questionnaire for oral hygiene practices. Data were analyzed using ANOVA, repeated-measures ANOVA, Chi-square, McNemar–Bowker, and multivariable linear regression.

**Results:**

Of 303 enrolled participants, 261 completed the study (dropout 13.9%). At 12 months, both gain- and loss-framed groups showed significantly lower PI scores compared to control (*p*-value = 0.012 and 0.03, respectively), with no difference between intervention groups. PI decreased by 28.46% (gain-framed) and 26.87% (loss-framed). Multivariable regression analysis confirmed gain-framed (B = − 0.429, *p*-value < 0.001) and loss-framed messaging (B = − 0.330, *p*-value < 0.001) as independent predictors of lower 12-month PI. GI and OHIP-5 scores showed no significant improvements in intervention groups; however, OHRQoL worsened in control group over time (*p*-value = 0.013, η2p = 0.12). Daily toothbrushing frequency was significantly higher in intervention groups at 12 months (*p*-value = 0.026), with no change in flossing.

**Conclusions:**

SMS-based oral health education improved plaque control and toothbrushing frequency among refugees, regardless of framing. However, SMS alone was insufficient to improve gingival health or OHRQoL, highlighting the need to combine mHealth with clinical care.

**Trial registration:**

ClinicalTrials.gov, TRN: NCT06649760, Registration date:12 October 2024.

**Supplementary Information:**

The online version contains supplementary material available at 10.1186/s12903-026-09252-z.

## Introduction

Sudan’s internal conflict has created one of the fastest-growing humanitarian and displacement crises in the world. Since April 2023, millions of Sudanese have been forced to flee their homes, with Egypt being their primary destination. By the end of January 2025, the total number of newly arrived Sudanese in Egypt reached more than 1.5 million, of which 759,855 individuals formally registered as refugees with the United Nations High Commissioner for Refugees (UNHCR) [1]. This massive displacement has made Sudanese citizens the largest refugee and migrant population in Egypt [2]. Refugees, in general, often face numerous health challenges, including oral health problems. Studies assessing refugees’ oral health consistently reported a higher prevalence of oral diseases and lower utilization of dental services compared to host populations [3]. Refugees typically seek oral health care only when there is an urgent need such as pain, especially with the lack of knowledge of the dental healthcare system in the host country [3, 4]. Consequently, oral health represents one of the urgent health care needs among this population, as untreated oral conditions, such as dental caries, can interfere with everyday activities including talking, eating, and sleeping, thereby reducing the overall health-related quality of life (OHRQoL) [5, 6].

Health education is a well-established and cost-effective method for preventing oral diseases, with effective removal of bacterial plaque being the gold standard for preventing most oral diseases [7]. However, traditional multi-visit educational programs are often impractical for refugees due to financial constraints and limited access to transportation [3]. Mobile health (mHealth), especially with the growing number of mobile phone users, offers a promising strategy to overcome these barriers and reach populations in remote and low-resource areas or where there are obstacles to access healthcare services [8]. By mid-2020, it was estimated that 96.7% of the world’s population was living within reach of a cellular network; 99.6% in developed and 96.1% in developing countries. Moreover, the use of Short Message Service (SMS) for targeted health education has been widely studied and shown to be a useful tool [9, 10]. An important aspect of designing health education messages is message framing. Messages can be framed in two different ways. Gain-framed messages emphasize the benefits of a behavior (e.g., “Flossing improves your oral health”), while loss-framed messages highlight the negative consequences of neglecting a behavior (e.g., “Not flossing can cause gingivitis”) [11]. The theoretical background behind message framing is prospect theory, which suggests that people are risk-seeking in the domain of losses and risk-averse in the domain of gains. This means that when decisions involve potential benefits or gains, people tend to avoid risky or uncertain outcomes, on the contrary, when decisions involve potential negative outcomes or losses, individuals may be more willing to accept risky or uncertain outcomes [12].

Despite the potential of framed SMS-based interventions, their effectiveness in improving the oral health of refugees populations has not yet been explored. Importantly, refugees represent a highly vulnerable group that exists across many different country contexts and health systems, making evidence-based, scalable, and low-cost interventions particularly valuable. Therefore, this study aimed to evaluate the impact of an oral health education intervention delivered via gain- and loss-framed mobile text messages on the oral health status, OHRQoL, and oral hygiene practices of a sample of Sudanese refugees residing in Egypt. The null hypothesis for this trial was that there would be no significant difference in oral health outcomes between refugees who receive framed oral health SMS messages and those who did not over a 12-month period.

## Methodology

### Design and setting

This study was conducted as a randomized, controlled, parallel-designed clinical trial with a 6- and 12 -month follow-up period. It took place between November 2024 and December 2025 at the Caritas Egypt Refugees office in Alexandria, Egypt. Participation was voluntary, using snowball and chain referral sampling techniques suitable for hard-to-reach populations [13].

### Participants

Participants were eligible if they were Sudanese refugees aged 18 years and above, residing in Alexandria, Egypt, for at least six months, able to read, and owning a mobile phone to receive text messages. Refugees were excluded if they were planning to relocate during the one-year study period or if they were currently participating in any oral health education or promotion programs.

### Ethical considerations

Ethical approval was granted by the Research Ethics Committee, Faculty of Dentistry, Alexandria University, Egypt (#0930–06/2024) prior to any research activities. The trial was registered on ClinicalTrials.gov in June 2024 and reported in accordance with the Consolidated Standards of Reporting Trials (CONSORT) guidelines. An information sheet and consent form to participate in the study were also secured. Confidentiality of participants’ data was ensured and access to data was restricted to the research team for research purposes only.

### Study procedures

After eligibility assessment and informed consent, participants completed the baseline clinical examination and the baseline questionnaire assessment (T0). They then attended a standardized oral health education session before being randomized to one of the three study groups. Following randomization, participants in the intervention groups received the framed SMS messages over the 12-month study period, while the control group received no SMS intervention. Clinical examinations and questionnaires were repeated at 6 months (T1) and 12 months (T2).

### Baseline education session

All participants received a brief 15-min standardized oral health education session in groups of ten. The session included a 10-min PowerPoint presentation followed by a 5-min question-and-answer period. The presentation covered common oral diseases, with emphasis on dental caries, chronic gingivitis, and periodontitis, including their causes, signs, and symptoms. Basic oral hygiene recommendations were also provided, including toothbrushing with fluoride toothpaste, flossing, and reducing sugary food intake. The sessions were conducted in neutral, factual language without framing elements, no gain- or loss-framed statements. The material was delivered using standardized slides and consistent wording by the same researcher (DA) to ensure uniformity across all groups.

### Intervention

The intervention included 24 gain-framed messages to Group 1 and 24 loss-framed messages to Group 2 (supplementary material 2). Messages had similar content but were framed differently; gain-framed messages focused on the benefits of adherence to oral hygiene, while loss-framed messages focused on the disadvantages of non-adherence to oral hygiene. The messages were sent once every two weeks, simultaneously to both intervention groups. Five experts and an educational psychologist evaluated the content validity of the SMS frameworks using the content validity index, calculating item-level (I-CVI) and scale-level indices (S-CVI/UA and S-CVI/Ave) [14]. The I-CVI ranged from 0.80 to 0.98, indicating strong agreement on the relevance of each item. The S-CVI/UA based on universal agreement was 0.85, and the average S-CVI (S-CVI/Ave) was 0.94. These results demonstrate that the SMS frameworks were considered highly relevant and appropriate by the experts. We anticipated that some refugees might block mass media messaging or advertising messages; therefore, the messages were sent with a registered number that was given to the refugees. In addition, to ensure that the messages were received, a researcher called the refugees randomly to enquire if they had received them. The control group received no interventions.

### Outcomes assessment

Outcome measures were assessed at baseline (T0), 6 months (T1), and 12 months (T2) using clinical examinations and a self-administered questionnaire. Study outcomes included changes in oral health status (clinical indices), OHRQoL, and oral hygiene practices.

#### Dental examination

Oral examination was carried out by a well-trained dentist (DA) and three interns. The interns were trained and calibrated for assessment of the study variables. Inter- and intra-examiner reliability were calculated separately for each oral index. For PI, the inter-examiner and intra-examiner kappa values were 0.81 and 0.85, respectively. For GI, the inter-examiner and intra-examiner kappa values were 0.74 and 0.79, respectively. These values indicated good-to-excellent reliability between examiners and across time [15]. All refugees were examined while seated in a regular chair, using flat disposable dental mirrors and blunt-tipped dental probes using a headlight for better illumination. In this study, oral health status was evaluated using Silness and Loe Plaque index (PI) [16, 17] and Löe and Silness Gingival index (GI) [18, 19]. PI assessed the thickness of plaque at the gingival area of 6 index teeth (1.6, 1.2, 2.4, 3.6, 3.2 and 4.6). Each surface (buccal, lingual, mesial, distal) was scored based on the following criteria: 0 for no plaque; 1 for a film of plaque detectable only by running a probe across the tooth surface; 2 for moderate soft deposits visible to the naked eye, accumulating within the gingival pocket or on the tooth; and 3 for an abundance of soft matter filling the area between the gingival margin and the tooth surface. The scores from the four areas of the tooth were added and divided by four to give the mean for this tooth. Then, the scores of the examined teeth were added and divided by the number of examined teeth to get the Plaque Index value. As for GI, it assessed the gingival status of the same six index teeth. Each surface (buccal, lingual, mesial, distal) was scored according to the following criteria: 0 for normal gingiva with no inflammation; 1 for mild inflammation characterized by slight color change and mild edema with no bleeding on probing; 2 for moderate inflammation characterized by redness, edema, and bleeding on probing; and 3 for severe inflammation characterized by marked redness and edema, ulceration, and tendency toward spontaneous bleeding. Similar to PI calculation, the mean GI score per tooth was calculated by averaging the scores of the four examined surfaces, and the overall GI score per participant was obtained by averaging scores across examined teeth.

#### Oral health-related quality of life

OHRQoL was measured by the 5-item Oral Health Impact Profile (OHIP) questionnaire, which has been translated and validated into Arabic. Its reliability and validity were tested and yielded good results, with Cronbach's alpha of 0.78 and an intraclass correlation coefficient (ICC) agreement of 0.88 [20]. The OHIP-5 assesses how patients are affected by oral disorders. Participants rate the frequency of experiencing oral problems in the last month, with responses on a 5-point Likert scale: 0 – never, 1 – hardly ever, 2 – occasionally, 3 – fairly often, and 4 – very often. The total OHIP-5 score is obtained by summing all responses with scores ranging from 0 to 20, with higher scores indicating worse OHRQoL.

#### Sociodemographic and oral hygiene practices assessment

Sociodemographic characteristics, access to oral healthcare, and oral health-related practices were assessed using a structured questionnaire adapted from previously published studies [21, 22]. The items were selected and combined to fit the objectives of the current study. The final questionnaire version used in this study is provided as Supplementary Material 1. The questionnaire consisted of two sections. The first section collected sociodemographic data including sex (male, female), age, marital status (married, unmarried), educational level (no formal education, secondary or less, or university and higher), and income per month (< 3000 EGP, 3000–6000 EGP, > 6000 EGP) [23]. The second section assessed whether refugees had access to primary oral healthcare (yes, no), oral health-related practices including toothbrushing frequency (at least once daily, few times per week, rarely/never), flossing (at least once daily, few times per week, rarely/never), and frequency of sugary snacking (at least once daily, few times per week, rarely/never). The questions were first developed in English, translated into Arabic, and then back-translated to English to confirm accuracy. The questionnaire was evaluated for content validity, with I-CVI values ranging from 0.81 to 1.00, S-CVI/UA of 0.86, and S-CVI/Ave of 0.97, indicating high relevance and appropriateness. The questionnaire was pilot-tested with 30 refugees to ensure clarity. Data from the pilot test were excluded from the final analysis.

### Sample size estimation

According to Divdar et al. [24], the mean ± SD dental plaque score among women who received gain-framed messages was 19.71 ± 9.71, those who received loss-framed messages was 23.67 ± 10.72, and those who did not receive any messages was 36.87 ± 11.74. Assuming 80% study power and 5% alpha error, based on comparison of group means, using two-tailed test, the minimum required sample size was 91 refugees per group increased to 101 to make up for losses. The total required sample size = number of groups × number per group = 3 × 101 = 303 refugees. Sample size was calculated using the MedCalc Statistical Software version 19.0.5 (MedCalc Software bvba, Ostend, Belgium; https://www. medcalc.org; 2019).

### Randomization, allocation concealment, and blinding

Following completion of the baseline assessment and standardized oral health education, eligible participants were randomly allocated in a 1:1:1 ratio to one of three groups: gain-framed messages, loss-framed messages, or control (no messages). Randomization was performed in blocks of 12 using a computer-generated list to reduce bias and achieve balance in the allocation of participants to interventions, increasing the probability that each arm will contain an equal number of individuals by sequencing participant assignment by block [25]. The randomization sequence was generated and managed by an independent researcher not involved in data collection or data analysis. Allocation was implemented electronically via the messaging system, and the team delivering the messages had no influence on group assignment. Outcome assessors and data analysts were blinded to groups allocation. Although participants were aware of whether they received text messages or not, they were not informed about the study design, hypotheses, or specific framing purposes.

#### Data analysis

Statistical analysis was conducted using SPSS 29.0 (SPSS Inc., IBM). Continuous data were presented as mean and standard deviation, while categorical data were presented as frequencies and percentages. The normality of continuous variables was assessed using the Shapiro–Wilk test and visual inspection of Q-Q plots. Although formal normality tests can be oversensitive in larger samples, the variables were considered approximately normally distributed. Furthermore, the Central Limit Theorem (*n* > 50 per group) supported the use of parametric procedures [26, 27]. Baseline homogeneity across the three groups was confirmed using One-way Analysis of Variance (ANOVA) for continuous variables and the Chi-square test for categorical variables, with a *P*-value > 0.05 indicating baseline comparability. Between-group differences at each follow-up point were examined using One-way ANOVA, with Tukey’s HSD post-hoc test applied when significant differences were found. Changes in Plaque Index, Gingival Index, and OHIP-5 scores over time within groups were analyzed using repeated-measures ANOVA. Percentage change from baseline to 12 months was calculated for each variable using the formula:

$$\begin{aligned}\%\;\mathrm{Change}&=\frac{12-\mathrm{month}\;\mathrm{mean}-\mathrm{Baseline}\;\mathrm{mean}}{\mathrm{Baseline}\;\mathrm{mean}}\\&\times100\end{aligned}$$

Negative values indicated a reduction from baseline, while positive values indicated an increase from baseline. The assumption of sphericity was assessed using Mauchly’s test. In instances where the assumption was violated (*P* < 0.05), degrees of freedom were adjusted using the Greenhouse–Geisser correction to ensure the validity of the F-statistic. Post-hoc longitudinal pairwise comparisons were conducted using Bonferroni correction for multiple comparisons. To further assess factors independently associated with 12-month plaque index scores and control for potential confounding variables, multivariable linear regression analysis was performed including age, gender, study group, education, income, and baseline plaque index score as independent variables. Oral hygiene practices were analyzed as categorical variables. Pearson Chi-square tests were used to compare their distribution between study groups at each time point (baseline, 6 months, and 12 months), while within-group changes between baseline and 12 months were assessed using the McNemar–Bowker test for paired categorical data. Analyses were performed according to a per-protocol approach, including only participants who completed the follow-up assessments and had complete outcome data. A two-sided *P*-value of < 0.05 was considered statistically significant.

#### Benefits/harms

Benefits from the study included: (1) Improved oral hygiene practices as a result of receiving targeted interventions (SMS-based messages) and (2) Improved OHRQoL scores. There were no anticipated or observed harms associated with participation in this trial.

## Results

**Fig. 1:**
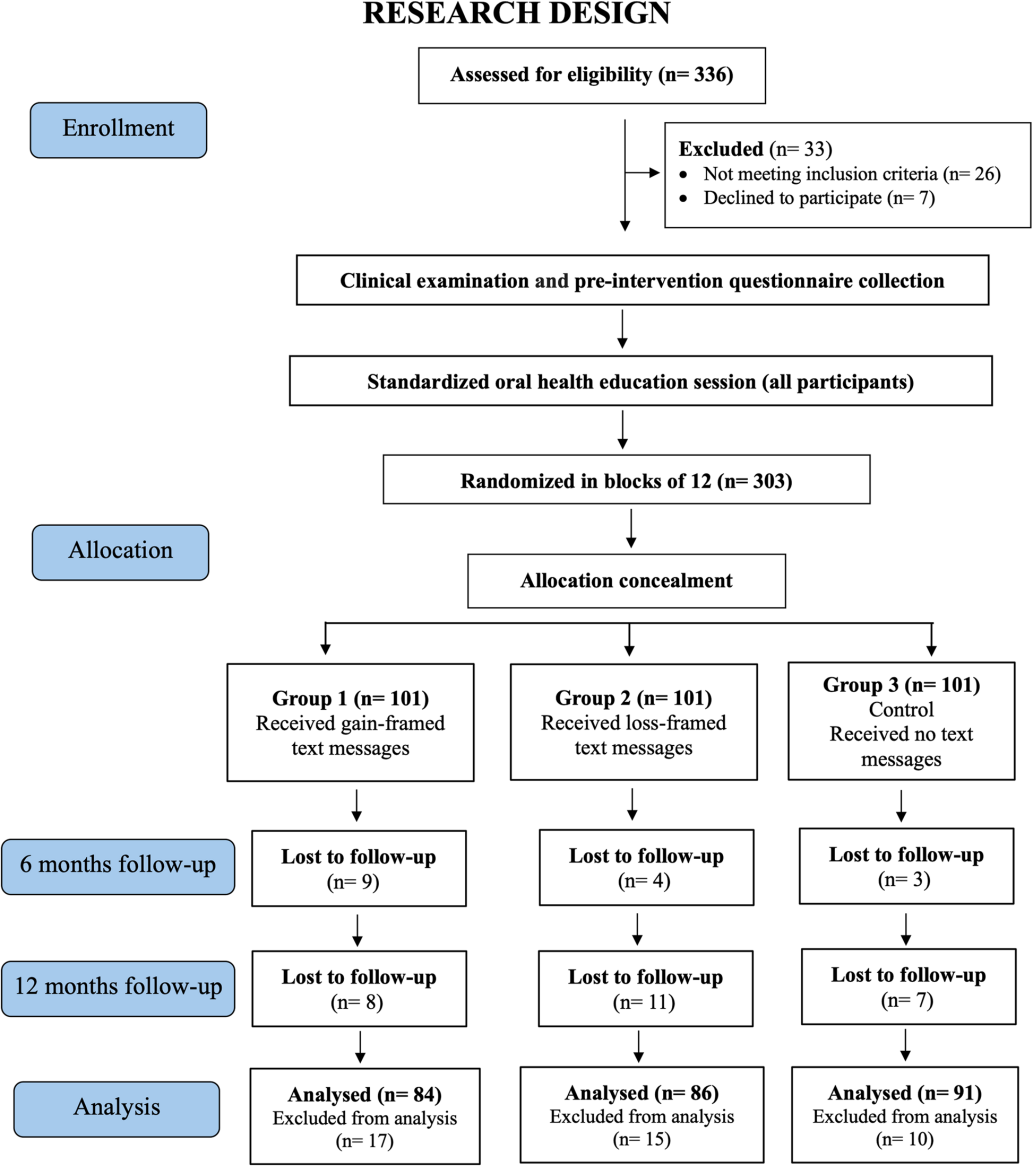
CONSORT flowchart of the progress throughout the 12-month follow-up study

Of the 303 participants enrolled, 261 (86.1%) completed the study, resulting in a dropout rate of 13.9% at 12-month follow-up (Fig. 1). The reasons for this dropout included relocation and the inability to attend follow-up visits. Baseline sociodemographic and clinical characteristics of the participants are presented in Table 1. Participants who were lost to follow-up were compared with those who completed the study and showed no statistically significant differences in baseline sociodemographic or clinical characteristics. No statistically significant differences were observed among the gain-framed, loss-framed, and control groups with respect to baseline sociodemographic, behavioral, or clinical characteristics (*P* > 0.05), indicating baseline comparability between groups. The mean ± SD age of the total sample was 44.7 ± 13.8 years, and females constituted 66.6% of the participants. Most participants were married (74.9%) and nearly half had attained university education or higher (51.9%). More than half of the participants (60.1%) reported a monthly income of < 3000 EGP and the majority (80.8%) reported that they had no access to primary oral healthcare. Regarding oral health practices, approximately one-third of participants (37.2%) reported brushing their teeth at least once daily, while almost a similar proportion (33.7%) reported consuming sugary snacks at least once daily. In contrast, 23.4% reported rarely or never brushing their teeth, and nearly all participants (88.2%) reported rarely or never using dental floss. The mean ± SD PI, GI, and OHIP-5 scores for the total sample at baseline were 1.30 ± 0.77, 1.13 ± 0.64, and 8.89 ± 3.52, respectively.

**Table 1:** Baseline sociodemographic characteristics, oral health practices, oral health status, and OHRQoL of study participants

Variable	Gain-framed group (*n* = 84)	Loss-framed group (*n* = 86)	Control group (*n* = 91)	Test statistic (df)	*P*-value
Age					
Mean (SD)	46.06 (14.31)	44.60 (14.51)	43.47 (12.33)	0.65 (2, 258)	0.47^#^
Gender, n (%)					
Male	35 (41.7)	26 (30.2)	26 (28.6)	3.93 (2)	0.14^Δ^
Female	49 (58.3)	60 (69.8)	65 (71.4)		
Marital status, n (%)					
Married	67 (79.8)	65 (75.6)	63 (69.2)	2.62 (2)	0.27^Δ^
Unmarried	17 (20.2)	21 (24.4)	28 (30.8)		
Educational level, n (%)					
No formal education	11 (13.6)	12 (14.3)	14 (15.7)	1.94 (4)	0.65^Δ^
Secondary or less	32 (39.5)	24 (28.6)	29 (32.6)		
University and higher	38 (46.9)	48 (57.1)	46 (51.7)		
Income level, n (%)					
< 3000 EGP	51 (60.7)	54 (62.8)	51 (56.7)	1.59 (4)	0.81^Δ^
3000–6000 EGP	27 (32.1)	23 (26.7)	30 (33.3)		
> 6000 EGP	6 (7.2)	9 (10.5)	9 (10.0)		
Access to primary oral healthcare, n (%)					
Yes	14 (16.7)	17 (19.8)	19 (20.9)	0.52 (2)	0.77^Δ^
No	70 (83.3)	69 (80.2)	72 (79.1)		
Toothbrushing frequency, n (%)					
At least once daily	31 (36.9)	32 (37.2)	34 (37.4)	0.014 (4)	1.00^Δ^
Few times per week	33 (39.3)	34 (39.5)	36 (39.5)		
Rarely/Never	20 (23.8)	20 (23.3)	21 (23.1)		
Dental flossing frequency, n (%)					
Once daily	2 (2.4)	3 (3.5)	4 (4.4)	1.05 (4)	0.90^Δ^
Few times per week	7 (8.3)	6 (7.0)	9 (9.9)		
Rarely/Never	75 (89.3)	77 (89.5)	78 (85.7)		
Sugary snacking frequency, n (%)					
At least once daily	25 (29.8)	33 (38.4)	30 (33.0)	1.46 (4)	0.83^Δ^
Few times per week	45 (53.6)	40 (46.5)	46 (50.5)		
Rarely/Never	14 (16.6)	13 (15.1)	15 (16.5)		
Baseline Plaque index					
Mean (SD)	1.34 (0.79)	1.33 (0.74)	1.23 (0.78)	0.52 (2, 258)	0.60^#^
Baseline Gingival index					
Mean (SD)	1.12 (0.62)	1.15 (0.65)	1.12 (0.66)	0.09 (2, 258)	0.91^#^
Baseline OHIP-5 score					
Mean (SD)	8.62 (3.36)	9.02 (3.42)	9.01 (3.71)	0.22 (2, 258)	0.82^#^

^#^:*P*-values derived from one-way ANOVA; test statistics represent *F* values with corresponding between-group and within-group degrees of freedom

^Δ^: *P*-values derived from Pearson Chi-square tests; test statistics represent χ2 values with corresponding degrees of freedom. *P* < 0.05 was considered statistically significant

### Plaque index, gingival index, and OHIP-5 scores over time

Longitudinal changes in PI, GI, and OHIP-5 scores are summarized in Table 2. Intergroup comparisons showed no statistically significant differences in PI scores at baseline or at 6 months. However, a statistically significant difference was observed at 12 months among the three groups (F (2, 258) = 4.785, *P* = 0.009, η^2^p = 0.035). Post-hoc Tukey HSD analysis showed that both the gain-framed and loss-framed groups had significantly lower PI scores than the control group (*P* = 0.012 and *P* = 0.03, respectively), with no significant difference between the two intervention groups.

**Table 2:** Intragroup and intergroup comparisons of Plaque index, Gingival index, and OHIP-5 scores in control and intervention groups at baseline, 6 and 12 months after intervention (*N* = 261)

Variable	Gain-framed (*n* = 84)	Loss-framed (*n* = 86)	Control (*n* = 91)	Between-group analysis	
				F (df)^a^	*P*-value, η^2^p
Plaque index (mean ± SD)					
Baseline	1.33 ± 0.79	1.34 ± 0.74	1.23 ± 0.78	0.519 (2, 258)	0.595, 0.001
6 months	1.27 ± 0.78	1.26 ± 0.74	1.24 ± 0.73	0.054 (2, 258)	0.948, < 0.001
12 months	0.93 ± 0.72	0.98 ± 0.75	1.26 ± 0.74	4.785 (2, 258)	0.009*, 0.035
% change	−28.46	−26.87	2.44		
Within-group analysis					
F (df)^b^	56.275 (1.14, 94.51)	54.869 (1.33, 112.71)	2.125 (1.24, 111.81)		
*P*-value, η^2^p	< 0.001*, 0.386	< 0.001*, 0.345	0.15, 0.034		
Gingival index (mean ± SD)					
Baseline	1.12 ± 0.62	1.15 ± 0.63	1.12 ± 0.62	0.091 (2, 258)	0.913, 0.001
6 months	1.11 ± 0.61	1.14 ± 0.64	1.12 ± 0.63	0.027 (2, 258)	0.973, < 0.001
12 months	1.09 ± 0.59	1.13 ± 0.62	1.14 ± 0.64	0.162 (2, 258)	0.851, 0.002
% change	−3.59	−3.45	1.79		
Within-group analysis					
F (df)^b^	3.026 (1.21, 100.61)	0.806 (1.70, 144.8)	1.611 (1.43, 128.56)		
*P*-value, η^2^p	0.077, 0.035	0.431, 0.009	0.208, 0.04		
OHIP-5 (mean ± SD)					
Baseline	8.62 ± 3.36	9.02 ± 3.42	9.01 ± 3.71	0.406 (2, 258)	0.816, 0.002
6 months	8.57 ± 3.89	8.96 ± 3.91	9.07 ± 3.69	0.588 (2, 258)	0.745, 0.002
12 months	8.50 ± 3.92	8.92 ± 3.52	9.17 ± 3.80	0.833 (2, 258)	0.659, 0.006
% change	−1.39	- 1.11	1.78		
Within-group analysis					
F (df)^b^	2.371 (1.87, 154.28)	1.326 (1.71, 145.77)	5.269 (1.31, 118.17)		
*P*-value, η^2^p	0.097, 0.028	0.268, 0.015	0.013*, 0.12		

^a^: Intergroup comparisons were performed using one-way ANOVA

^b^: Intragroup comparisons were performed using repeated-measures ANOVA with Greenhouse–Geisser correction. df: degree of freedom; η^2^p: partial eta squared

^*^: statistically significant at *P* < 0.05. Post-hoc Tukey's HSD for intergroup comparisons of Plaque index scores at 12 months showed a statistically significant difference between the gain-framed and control groups (*P* = 0.012) and loss-framed and control groups (*P* = 0.03), with no significant differences between the gain- and loss-framed groups

Within-group analysis revealed significant reductions in PI scores over time in both the gain-framed (F (1.14, 94.51) = 56.275, *P* < 0.001, η^2^p = 0.386) and loss-framed (F (1.33, 112.71) = 54.869, *P* < 0.001, η^2^p = 0.345) groups, corresponding to mean reductions of − 28.46% (from 1.33 ± 0.79 to 0.93 ± 0.72) and − 26.87% (from 1.34 ± 0.74 to 0.98 ± 0.76), respectively. Conversely, the control group showed non-significant change over time (F (1.24, 111.81) = 2.125, *P* = 0.15, η^2^p = 0.034). Furthermore, intragroup Bonferroni-corrected post-hoc tests (Table 3) confirmed that in both intervention groups, PI scores significantly decreased across all time intervals (Baseline vs 6 months, Baseline vs 12 months, and 6 months vs 12 months; all *P* < 0.001), indicating a continuous improvement throughout the study period.

**Table 3:** Within-group comparisons of mean Plaque Index scores and OHIP-5 scores across follow-up time points using Bonferroni-corrected post-hoc tests

	Time intervals		Mean difference (SE)	95% CI	*P*-value
Plaque index scores in gain-framed group	Baseline	6 months	0.064 (0.017)	0.023, 0.106	< 0.001*
		12 months	0.407 (0.054)	0.274, 0.540	< 0.001*
	6 months	12 months	0.343 (0.048)	0.226, 0.459	< 0.001*
Plaque index scores in loss-framed group	Baseline	6 months	0.093 (0.023)	0.037, 0.149	< 0.001*
		12 months	0.322 (0.045)	0.212, 0.433	< 0.001*
	6 months	12 months	0.229 (0.034)	0.148, 0.312	< 0.001*
OHIP-5 scores in control group	Baseline	6 months	- 0.077 (0.047)	−0.193, 0.039	0.326
		12 months	- 0.352 (0.081)	−0.585, −0.119	0.001*
	6 months	12 months	- 0.275 (0.072)	−0.450, −0.100	< 0.001*

*SE* standard error, *CI* confidence interval

^*^: statistically significant at *P* < 0.05. *P*-values and confidence intervals were Bonferroni-adjusted for multiple comparisons

**Fig. 2:**
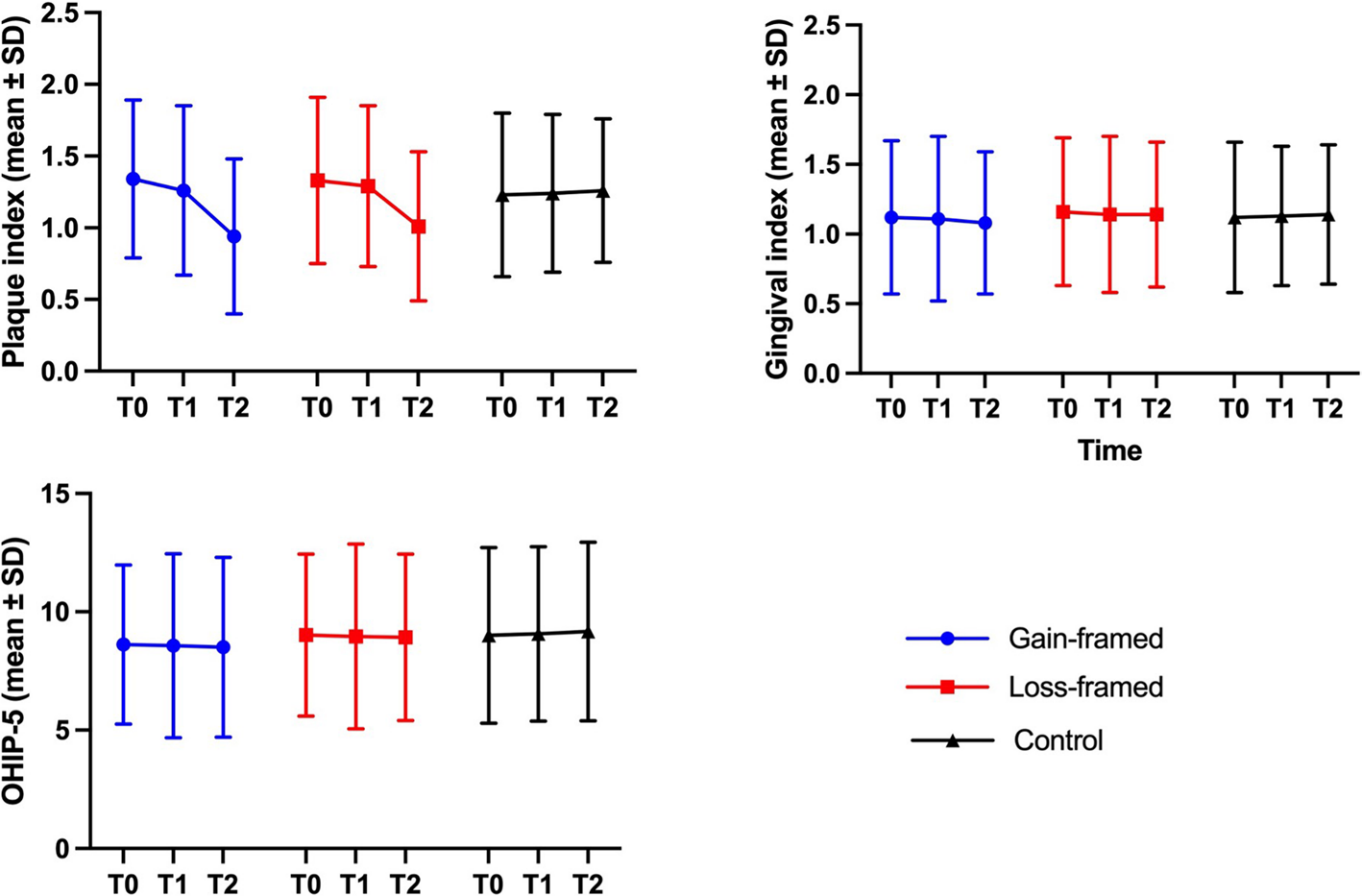
Longitudinal changes in Plaque Index (PI), Gingival Index (GI), and OHIP-5 scores across the three study groups

For the GI, neither the intragroup nor the intergroup comparisons demonstrated any statistically significant differences over the 12 months follow-up. While small reductions were observed in the gain-framed (− 3.59%) and loss-framed (− 3.45%) groups and a slight increase in the control group (1.79%), these differences were not statistically significant. Similarly, the gain- and loss-framed groups showed no significant change in OHIP-5 scores, despite small mean reductions of − 1.39% and − 1.11%, respectively. In contrast, the control group demonstrated a statistically significant increase in OHIP-5 scores over time (F (1.31, 118.17) = 5.269, *P* = 0.013, η^2^p = 0.12), indicating a deterioration in OHRQoL over time in the absence of intervention. Post-hoc comparisons showed that this worsening was significant between 6 and 12 months (*P* < 0.001) and between baseline and 12 months (*P* = 0.001). No statistically significant intergroup differences in OHIP-5 scores were observed at any follow-up time point. Longitudinal changes in PI, GI, and OHIP-5 scores across the three groups are illustrated in Fig. 2. Multivariable linear regression analysis was performed to identify factors independently associated with 12-month Plaque Index scores (Table 4). After adjustment for age, gender, education, income, and baseline Plaque Index score, participants in both the gain-framed and loss-framed groups had significantly lower 12-month Plaque Index scores compared with the control group (B = − 0.429, 95% CI: − 0.539 to − 0.318, *P* < 0.001 and B = − 0.330, 95% CI: − 0.439 to − 0.221, *P* < 0.001, respectively). Baseline Plaque Index score was also a significant predictor of 12-month Plaque Index score (B = 0.689, 95% CI: 0.607–0.771, *P* < 0.001). The model explained 63.2% of the variance in 12-month Plaque Index scores (adjusted R2 = 0.632).

**Table 4:** Multivariable linear regression analysis of factors associated with 12-month Plaque Index scores

Independent variable	B (95% CI)	*P*-value
Age	0.005 (−0.001, 0.007)	0.069
Gender		
Male	0.098 (−0.004, 0.200)	0.180
Female	Reference	
Study group		
Gain-framed	−0.429 (−0.539, −0.318)	< 0.001*
Loss-framed	−0.330 (−0.439, −0.221)	< 0.001*
Control	Reference	
Education		
No formal education	0.060 (−0.095, 0.215)	0.449
Secondary or less	0.024 (−0.085, 0.133)	0.670
University or higher	Reference	
Income		
< 3000	0.021 (−0.148, 0.176)	0.817
3000–6000	0.006 (−0.177, 0.165)	0.943
> 6000	Reference	
Baseline score	0.689 (0.607, 0.771)	< 0.001*

*B* regression coefficient, *CI* Confidence Interval; Adjusted R^2^ = 0.632

^*^statistically significant at *p* < 0.05

### Oral hygiene practices over time

Table 5 summarizes changes in toothbrushing and flossing practices across and within the study groups over time. For toothbrushing frequency, no statistically significant differences were observed between groups at baseline (*P* = 1.00) or at 6 months (*P* = 0.565). However, a statistically significant difference was observed at 12 months (χ^2^ = 11.027, *P* = 0.026). At this final time point, a higher proportion of participants in the gain-framed (54.8%) and loss-framed (54.7%) groups reported brushing at least once daily compared to the control group (38.5%). Within-group analyses demonstrated significant improvements in toothbrushing frequency between baseline and 12 months in both the gain-framed (χ2_B_ = 24.389, *P* < 0.001) and loss-framed groups (χ2_B_ = 23.908, *P* < 0.001), whereas no significant change was observed in the control group (χ2_B_ = 1.932, *P* = 0.368). For flossing frequency, no statistically significant differences were observed between groups at any time point (all *P* > 0.05). Similarly, within-group analyses showed no statistically significant changes in flossing behavior between baseline and 12 months in the gain-framed, loss-framed, or control groups (all *P* > 0.05).

**Table 5:** Oral hygiene practices over time within and across study groups [n, (%)]

Practice and time point	Frequency category	Gain-framed (*n* = 84)	Loss-framed (*n* = 86)	Control (*n* = 91)	X^2^	*P*-value
Toothbrushing						
Baseline	At least once daily	31 (36.9)	32 (37.2)	34 (37.4)	0.014	1.00
	Few times per week	33 (39.3)	34 (39.5)	36 (39.5)		
	Rarely/Never	20 (23.8)	20 (23.3)	21 (23.1)		
6 months	At least once daily	36 (42.9)	39 (45.3)	35 (38.5)	2.956	0.565
	Few times per week	37 (44.0)	33 (38.4)	36 (39.5)		
	Rarely/Never	11 (13.1)	14 (16.3)	20 (22.0)		
12 months	At least once daily	46 (54.8)	47 (54.7)	35 (38.5)	11.027	0.026*
	Few times per week	31 (36.9)	31 (36.0)	36 (39.5)		
	Rarely/Never	7 (8.3)	8 (9.3)	20 (22.0)		
Intragroup analysis (Baseline vs 12 months)						
X^2^_B_, *P*-value		24.389, < 0.001*	23.908, < 0.001*	1.932, 0.368	-	**-**
Flossing						
Baseline	At least once daily	2 (2.4)	3 (3.5)	4 (4.4)	1.054	0.902
	Few times per week	7 (8.3)	6 (7.0)	9 (9.9)		
	Rarely/Never	75 (89.3)	77 (89.5)	78 (85.7)		
6 months	At least once daily	3 (3.6)	3 (3.5)	4 (4.4)	0.639	0.959
	Few times per week	7 (8.3)	6 (7.0)	9 (9.9)		
	Rarely/Never	74 (88.1)	77 (89.5)	78 (85.7)		
12 months	At least once daily	4 (4.8)	4 (4.7)	4 (4.4)	1.102	0.894
	Few times per week	12 (14.3)	8 (9.3)	10 (11.0)		
	Rarely/Never	69 (81.0)	74 (86.0)	77 (84.6)		
Intragroup analysis (Baseline vs 12 months)						
X^2^_B_, *P*-value		4.778, 0.092	2.003, 0.268	1.10, 0.317	-	-

X^2^: Pearson Chi-square test for between-group comparisons at each time point; X^2^_B_: McNemar-Bowker Test for within-group comparisons between baseline and 12 months; *: statistically significant at *P* < 0.05

## Discussion

This 12-month randomized controlled trial demonstrated that both gain- and loss-framed SMS messages led to significant improvements in oral hygiene, as measured by reduction in plaque levels and increase in self-reported toothbrushing frequency, among a Sudanese refugee population living in Egypt. The study was designed to address the unpredictable and difficult circumstances faced by refugee populations, which make the implementation of oral health education programs requiring multiple visits difficult. Common barriers such as financial burdens, mobility issues, and limited access to transportation make traditional oral health programs challenging and unrealistic [28]. While this study used a longitudinal design with follow-up assessments at 6 and 12 months to evaluate impact on longer time intervals, the intervention itself needed to be minimally disruptive, adaptable and more flexible. Therefore, SMS-based messaging was chosen because it delivers consistent, low-cost oral health education directly to participants, maintaining intervention consistency without imposing the burden of repeated clinical visits beyond essential data collection points [8].

The results showed that both gain- and loss-framed messages were effective in lowering PI scores compared with the control group, with no significant difference between the two intervention types. This finding suggests that the framing style of motivational messages may be less important than the consistent reinforcement of oral hygiene practices, regardless of the tone. Although these results differ from the concept adopted by prospect theory, which postulates that gain-framed messages are more effective for promoting preventive behaviors [12], similar findings have been reported in previous studies. Khademian et al. [29] found that gain- and loss-framed SMS had similar impacts on the mothers’ knowledge and practices related to their children’s oral health. Also, Divdar et al. [24] demonstrated that periodic SMS reminders improved oral health behaviors and plaque levels among pregnant women, regardless of the type of message framing. Additionally, several contextual factors related to the refugee setting might explain the absence of a differential effect between gain- and loss-framed messaging. Refugee populations often experience chronic psychological stress, socioeconomic hardship, disrupted living conditions, and limited access to healthcare, all of which may reduce the cognitive and emotional processing differences typically expected between message-framing strategies [30, 31]. In such contexts, repeated oral health reminders and continuous behavioral reinforcement may be more influential than the specific emotional framing of the messages. In addition, variations in health literacy may have reduced participants’ sensitivity to subtle framing differences, leading both intervention types to function primarily as regular motivational cues rather than distinct persuasive strategies [32]. These findings may indicate that, within vulnerable and resource-limited populations, the frequency and consistency of health communication could play a more important role in behavior change than message framing itself. Furthermore, the association between the intervention and lower 12-month PI scores remained significant after adjustment for age, gender, education, income, and baseline plaque index scores, supporting the independent effect of the intervention on plaque reduction. The large effect sizes observed for plaque reduction support the potential value of the intervention in improving oral hygiene behaviors. Beyond message framing, baseline PI was the strongest independent predictor of 12-month outcomes, suggesting that refugees with poorer baseline oral health may require more intensive or personalized support.

Despite improvements in plaque control, no significant changes were observed in GI scores over time. Although this finding contrasts with the results of the systematic review by Toniazzo et al. [33], which found significant reductions in both plaque and gingival indices; however, this review reported high heterogeneity between studies and smaller effects on gingival inflammation. Likewise, Abdul Haq et al. [34] observed a significant reduction in the Approximal Plaque Index but not in the Papillary Bleeding Index in their digital application–based oral health program for parents, suggesting that plaque control improvements do not always translate into immediate reductions in gingival inflammation. The divergence between plaque and gingival improvements in the present study could be attributed to several biological and contextual factors. Inflammation resolution requires tissue recovery even after biofilm disruption, therefore, gingival healing sometimes lags behind plaque removal. Other factors, such as hormonal fluctuations and systemic diseases, or medication use, may also contribute to residual gingival inflammation independent of supragingival plaque reduction. Moreover, persistent subgingival plaque or calculus may remain inaccessible to regular toothbrushing and often require professional scaling and adjunctive treatment to achieve complete gingival health restoration [35]. Previous community-based interventions have highlighted that limited access to dental care and the absence of professional treatment during the intervention are some of the reasons for why their behavioral intervention was not effective [36]. This may partly explain the absence of significant gingival improvement, particularly given the high proportion of participants reporting limited access to primary oral healthcare. Similarly, OHIP-5 scores did not show significant improvement in either intervention group; however, the control group demonstrated a statistically significant deterioration in OHRQoL over time with a moderate effect size. This suggests that while the interventions did not enhance OHRQoL, they may have helped maintain stability over time, whereas the control group experienced gradual deterioration. The stable scores in the intervention groups may reflect the interventions’ role in supporting oral hygiene behaviors and preventing gradual deterioration of oral health in this vulnerable population who face multiple stressors. Nevertheless, the lack of overall improvement underscores the multifactorial nature of OHRQoL, which depends not only on hygiene behaviors but also on clinical management of underlying oral diseases, often limited by access to dental services [37]. These findings are consistent with Alrashdi et al. [38], who reported no significant improvements in clinical or OHRQoL of life among refugee children following a preventive outreach intervention, attributing this to persistent barriers to dental care and socioeconomic challenges, factors that may also have influenced the outcomes in the present study. As for oral hygiene practices, an increase in self-reported daily toothbrushing frequency was observed in both intervention groups at 12 months compared to the control group, consistent with the improvement in plaque scores. However, no improvement was observed in flossing frequency. This could be explained by the more complex nature of flossing that requires manual dexterity, higher levels of motivation, and could be hindered by the fear of bleeding [39, 40]. In addition, socioeconomic level could have an effect on the availability of flossing tools, which is relevant here, since more than half of participants (60.1%) reported having low income. Moreover, the lack of hands-on training or clinical reinforcement in this intervention likely limited behavioral adoption of flossing, which agrees with a review suggesting that oral health education programs should be supported by multiple other health promotion interventions since oral health education alone is of limited value [41]. Taken together, these findings suggest that although the intervention improved plaque control and toothbrushing practices, broader clinical and quality-of-life benefits remained limited without additional preventive or professional dental support. Therefore, future research should investigate the integration of mHealth interventions with periodic professional care to address hard-to-change outcomes such as gingival health and OHRQoL.

The study has some limitations. First, the use of snowball sampling may have introduced selection bias and may limit the representativeness of the sample. As a non-probability sampling method, this approach may affect the external validity of the findings, and therefore the results may not be fully generalizable to the broader refugee population. In addition, snowball sampling may preferentially recruit participants through existing social networks, potentially underrepresenting refugees with fewer social connections or different patterns of healthcare access. Therefore, the findings may be more reflective of socially connected refugee networks rather than the refugee population as a whole. However, this method is commonly recommended for hard-to-reach populations, and randomization within the sample helped minimize systematic differences between groups. Second, there is a potential for contamination, as all participants received an initial oral health education session. This was done for ethical reasons to ensure that no group, including the control, was deprived of basic oral health information, given the recognized vulnerability and limited access to health services among refugees. This standardized baseline education may also have attenuated differences between study groups and reduced the ability to isolate the independent effect of message framing itself, potentially contributing to the absence of differences observed between gain- and loss-framed interventions. Third, the assessment of oral hygiene behaviors (toothbrushing and flossing) relied on self-reported data, which is subject to information bias, including recall bias and social desirability bias. This may have led to an overestimation of favorable behaviors, particularly given the repeated contact with participants through SMS messages and follow-up assessments. Additionally, a Hawthorne effect may have occurred, as participants could have temporarily improved their behaviors due to awareness of being observed. Nevertheless, these effects are unlikely to fully explain the findings, as improvements were observed primarily in intervention groups despite identical baseline education and follow-up procedures. This clear differential effect indicates that while the baseline education and observation were constants, the framed messaging provided a unique, additive motivational boost that translated into superior behavioral outcomes. Another limitation of this study is the reliance on a per-protocol analysis rather than an intention-to-treat (ITT) analysis and the absence of formal sensitivity analyses to evaluate the impact of missing data. While an ITT approach is generally preferred to preserve the benefits of baseline randomization, a per-protocol approach was used because the primary longitudinal analyses relied on complete repeated clinical measurements collected across follow-up intervals. Although this strategy may introduce a risk of attrition bias, participants lost to follow-up did not differ significantly from completers in baseline sociodemographic or clinical characteristics, reducing concerns regarding systematic differences related to study retention. Nonetheless, because neither an ITT nor a sensitivity analysis was performed, the potential for bias cannot be entirely excluded, and the findings should be interpreted in the context of participants who completed the study. Moreover, the questionnaire did not include information on participants’ systemic diseases, medication use, or smoking status. This could have limited the ability to assess potential confounding factors that might have influenced gingival inflammation and healing processes, regardless of plaque levels. For instance, smoking may influence gingival bleeding responses, while systemic inflammatory conditions, medication use, and uncontrolled systemic diseases can affect periodontal healing and oral health–related quality of life [42, 43]. Consequently, residual confounding cannot be excluded, and interpretation of the nonsignificant findings observed for GI and OHIP-5 outcomes should be made cautiously. Furthermore, while the intervention was theoretically informed by message framing and prospect theory, psychological mediators such as motivation, perceived susceptibility, self-efficacy, and participant engagement with the SMS messages were not assessed. Therefore, the mechanisms underlying the observed behavioral improvements cannot be fully explained. Future studies should incorporate behavioral and psychological measures to better understand how framed mHealth interventions influence oral health behaviors among refugee populations. In addition, although phone calls were conducted to confirm SMS receipt, intervention adherence could not be objectively verified, as no measures were available to confirm whether participants read or engaged with the messages. Finally, the predominance of females (66.6%) may limit the generalizability of findings to male refugees; however, this distribution is aligned with reported demographic trends for Sudanese refugees in Egypt, where women and children comprised approximately 75% of the displaced population [44]. However, the study has several strengths. A key strength of this randomized controlled trial is its long-term, 12-month follow-up period, which provides evidence on the sustainability of the effects of mHealth intervention, moving beyond immediate post-intervention results. The low dropout rate enhances the reliability of the findings and minimizes potential attrition bias. Furthermore, the incorporation of objective clinical examinations (PI and GI) in addition to the self-reported practices offers a comprehensive and reliable assessment, verifying self-reported improvements with biological evidence and strengthening the study's conclusions.

## Conclusions

This study demonstrated that SMS-based oral health education, regardless of message framing, represents a feasible approach that improved plaque control and toothbrushing practices among refugees. However, this low-cost intervention alone was insufficient to improve gingival health or OHRQoL, suggesting that behavioral improvements may not necessarily translate into broader clinical benefits without integration with professional dental care. These findings underscore the need to integrate mHealth interventions within broader public health strategies and enhance access to professional dental care to achieve more comprehensive oral health improvements among vulnerable and displaced populations.

## Supplementary Information

Supplementary Material 1.

Supplementary Material 2.

## Data Availability

The datasets analysed during the current study are available from the corresponding author on reasonable request.

## Abbreviations

UNHCR: United Nations High Commissioner for Refugees
OHRQoL: Oral Health-related Quality of Life
PI: Plaque Index
GI: Gingival Index
OHIP: Oral Health Impact Profile
