# Supplementary material for: Effect of framed mHealth messages on oral hygiene and quality of life among Sudanese refugees in Egypt: a randomized controlled trial
Source: BMC Oral Health. 2026 Jul 17;26:1275. doi: 10.1186/s12903-026-09252-z (PMC13377808; doi:10.1186/s12903-026-09252-z)
Supplement: Supplementary file 1 — Supplementary Material 1. [file 12903_2026_9252_MOESM1_ESM.docx]

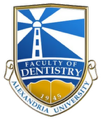


Dear Participant,

Thank you for taking the time to complete this questionnaire.

Your participation is completely voluntary, and all information you provide will be kept strictly confidential and used only for research purposes. Please answer the questions honestly and based on your actual experiences. Your responses are very important and will help improve oral health services provided for the community.

Thank you for your cooperation and support!

**I. Sociodemographic Information**

**01. What is your gender?**
a. Male
b. Female

**02. How old are you?**
_____ (years old)

**03. What is your marital status?**

a. Married
b. Unmarried

**04. What is your highest level of education completed?**

a. No formal education

b. Secondary education or less

c. University and higher

**05. What is your approximate monthly income?**

a. < 3000 EGP

b. 3000-6000 EGP

c. > 6000 EGP

**II. Oral-health related information**

**01. Do you have access to primary oral healthcare in Egypt?**

a. Yes

b. No

**02. How often do you brush your teeth?**a. At least once daily

b. Few times per week
c. Rarely/never

**03. How often do you use dental floss?**a. At least once daily

b. Few times per week
c. Rarely/never

**04. How often do you consume sugary foods or drinks?**

a. At least once daily

b. Few times per week
c. Rarely/never

**III. Oral-health related quality of life (OHIP-5)**

| **During the last month, how frequently have you experienced the following problems?** | Never | Hardly ever | Occasionally | Fairly often | Very often |
| --- | --- | --- | --- | --- | --- |
| 1- Have you had difficulty chewing any foods because of problems with your teeth, mouth, dentures, or jaw? |  |  |  |  |  |
| 2- Have you had painful aching in your mouth? |  |  |  |  |  |
| 3- Have you felt uncomfortable about the appearance of your teeth, mouth, dentures, or jaws? |  |  |  |  |  |
| 4- Have you felt that there has been less flavor in your food because of problems with your teeth, mouth, dentures, or jaws? |  |  |  |  |  |
| 5- Have you had difficulty doing your usual jobs because of problems with your teeth, mouth, dentures, or jaws? |  |  |  |  |  |
