# Supplementary material for: Effect of framed mHealth messages on oral hygiene and quality of life among Sudanese refugees in Egypt: a randomized controlled trial
Source: BMC Oral Health. 2026 Jul 17;26:1275. doi: 10.1186/s12903-026-09252-z (PMC13377808; doi:10.1186/s12903-026-09252-z)
Supplement: Supplementary file 2 — Supplementary Material 2. [file 12903_2026_9252_MOESM2_ESM.docx]

**Full set of gain-framed and loss-framed SMS intervention messages used in the study**

- **Participants received Arabic SMS messages. English translations are provided for reproducibility and theoretical interpretation.**

| **No.** | **Gain-framed message** | **Loss-framed message** |
| --- | --- | --- |
| 1 | Brushing your teeth twice a day can help you maintain a bright, healthy smile. | Neglecting to brush your teeth twice a day may result in a dull and unhealthy smile. |
| 2 | Brushing your teeth before bedtime helps remove bacteria and protect your teeth overnight. | Skipping brushing before bedtime may allow bacteria to remain longer and increase the risk of tooth damage. |
| 3 | Good oral hygiene helps reduce plaque buildup. | Poor oral hygiene can contribute to plaque accumulation. |
| 4 | Brushing your teeth thoroughly every morning and night helps maintain fresh breath and removes harmful bacteria. | Skipping your morning or nightly toothbrushing routine may cause bad breath and allow harmful bacteria to damage your teeth. |
| 5 | Using a soft-bristled toothbrush protects your enamel and massages your gums gently | Using a hard-bristled toothbrush may wear away your tooth enamel and damage your gums. |
| 6 | Brushing gently in circular motions for a full two minutes helps clean your teeth effectively while protecting your gums. | Brushing too hard or for less than two minutes could leave plaque behind and damage your gums. |
| 7 | Fluoride toothpaste strengthens your teeth and helps prevent cavity formation. | Not using fluoride toothpaste may make your teeth more susceptible to cavity formation. |
| 8 | Replacing your toothbrush every three months ensures effective cleaning. | Using an old toothbrush may reduce its effectiveness in removing plaque. |
| 9 | Using dental floss can help remove plaque from areas your toothbrush cannot reach. | Failing to floss may allow plaque to accumulate in hard-to-reach areas. |
| 10 | Cleaning your tongue can help eliminate bacteria and keep your breath fresh. | Neglecting to clean your tongue may lead to bacterial buildup and bad breath. |
| 11 | Replacing sugary snacks with healthier options, like fruits, can improve your oral health. | Regularly eating sugary snacks can damage your teeth. |
| 12 | Limiting sugary drinks helps maintain healthier teeth. | Frequent sugary drinks can damage teeth over time. |
| 13 | Drinking plenty of water helps wash away food particles and bacteria. | Not drinking enough water may allow food particles and bacteria to remain in your mouth longer. |
| 14 | Eating a balanced diet supports strong teeth and gums. | A poor diet can weaken your teeth and gums, leading to oral health issues. |
| 15 | Rinsing your mouth after meals can help remove food particles and prevent decay. | Failing to rinse your mouth after meals may allow food particles to cause decay. |
| 16 | Keeping hydrated helps maintain saliva flow, which protects your teeth. | Dehydration can reduce saliva flow, increasing your risk of tooth decay. |
| 17 | Choosing water and healthy snacks instead of sweets protects your enamel and keeps your teeth healthy. | Consuming sugary snacks and soft drinks constantly destroys your tooth enamel, leading to costly and painful tooth decay. |
| 18 | Healthy gums support strong teeth and a healthier smile. | Ignoring gum health can lead to inflammation and oral problems. |
| 19 | Healthy oral habits can help you feel more comfortable eating and speaking. | Poor oral health may interfere with eating and speaking comfortably. |
| 20 | Consistent brushing habits help maintain long-term oral health. | Inconsistent brushing habits may increase oral health problems over time. |
| 21 | Caring for your oral health is an important step toward better overall health. | Ignoring oral health may negatively affect your overall health. |
| 22 | Keeping your mouth clean prevents oral odors, helping you feel confident and comfortable when talking closely with family and friends. | Neglecting daily oral hygiene may contribute to bad breath, which can lead to discomfort and reduced confidence during social interactions. |
| 23 | Consistent, daily oral care preserves your natural teeth as you grow older, keeping your body healthy and saving you money. | Neglecting daily oral hygiene may increase the risk of early tooth loss, which can affect your nutrition and increase treatment needs over time. |
| 24 | Regular dental check-ups can catch issues early, keeping your mouth healthy. | Skipping professional cleanings may allow oral problems to progress before treatment. |
